# Bidirectional Association between Asthma and Irritable Bowel Syndrome: Two Population-Based Retrospective Cohort Studies

**DOI:** 10.1371/journal.pone.0153911

**Published:** 2016-04-19

**Authors:** Te-Chun Shen, Cheng-Li Lin, Chang-Ching Wei, Chia-Hung Chen, Chih-Yen Tu, Te-Chun Hsia, Chuen-Ming Shih, Wu-Huei Hsu, Fung-Chang Sung, Chia-Hung Kao

**Affiliations:** 1 Graduate Institute of Clinical Medicine Science, College of Medicine, China Medical University, Taichung, Taiwan; 2 Division of Pulmonary and Critical Care Medicine, Department of Internal Medicine, China Medical University Hospital, Taichung, Taiwan; 3 Management Office for Health Data, China Medical University Hospital, Taichung, Taiwan; 4 Children’s Hospital, China Medical University Hospital, China Medical University, Taichung, Taiwan; 5 Department of Health Services Administration, China Medical University, Taichung, Taiwan; 6 Faculty of Public Health, Mahidol University, Bangkok, Thailand; 7 Department of Nuclear Medicine and PET Center, China Medical University Hospital, Taichung, Taiwan; The Australian National University, AUSTRALIA

## Abstract

**Background:**

There is a demonstrated association between asthma and irritable bowel syndrome (IBS). In this study, we examined the bidirectional association between asthma and IBS using a nationwide database.

**Methods:**

We conducted two retrospective cohort studies using data obtained from the National Health Insurance of Taiwan. Study 1 included 29,648 asthma patients newly diagnosed between 2000 and 2010. Study 2 included 29,875 IBS patient newly diagnosed between 2000 and 2010. For each study, four subjects without asthma and IBS were selected, respectively, frequency-matched by sex, age, and the diagnosis date. All four cohorts were followed up until the end of 2011 to estimate incident IBS for Study 1 and incident asthma for study 2. Adjusted hazard ratios (aHRs) were estimated using the Cox proportional hazards model after controlling for sex, age and comorbidities.

**Results:**

The incidence of IBS was 1.89 times higher in the asthma cohort than in the comparison cohort (8.26 vs. 4.36 per 1,000 person-years), with an aHR of 1.57 [95% confidence interval (CI) = 1.47–1.68]. The aHRs remained significant in all subgroups measured by sex, age and the presence of comorbidities. In contrast, the incidence of asthma was 1.76 times higher in the IBS cohort than the comparison cohort (7.09 vs. 4.03 per 1,000 person-years), with an aHR of 1.54 (95% CI = 1.44−1.64). Similarly, aHRs remained significant in all subgroups measured by sex, age and the presence of comorbidities.

**Conclusion:**

The present study suggests a bidirectional association between asthma and IBS. Atopy could be a shared pathophysiology underlying this association, deserving a further investigation.

## Introduction

Asthma is a serious health problem affecting an estimated population of 300 million worldwide of all age groups. Asthma is defined based on characteristic symptoms and variation in expiratory airflow [1]. Patients with asthma suffer from respiratory symptoms and limited daily activities. An acute exacerbation of asthma may need urgent health care. Certain comorbidities commonly present in patients with asthma, such as gastroesophageal reflux disease (GERD), rhinitis, sinusitis, anxiety, and depression [2–5]. In addition, studies have demonstrated that asthma is associated with functional gastrointestinal disorders (FGIDs) due to the activation of the immune system [6, 7].

Irritable bowel syndrome (IBS) is a chronic FGID, which affects 10–15% of the general population, with a higher prevalence in women than in men [8]. The Rome III system is the most widely used criteria for the diagnosis of FGIDs, including for the diagnosis of IBS. Based on the Rome III system, patients fulfilling criteria of IBS for the last 3 months with symptom onset and for at least 6 months prior to diagnosis are diagnosed with IBS. Patients suffer from recurrent abdominal pain or discomfort for at least 3 days in a month in the past 3 months and have been associated with two or more of the following: 1. improvement with defecation, 2. onset associated with a change in frequency of stool, 3. onset associated with a change in form (appearance) of stool [9]. The pathophysiology of IBS is complex, involving the digestive organ dysmotility, bacterial flora alteration, visceral hypersensitivity, dysregulation of mucosal immune, and dysregulation between the central nervous system and enteric nervous system [10].

Immune activation has been associated with both asthma and IBS. The T-helper 2 (TH2)-type immune response is well-known predominant in patients with asthma [11]. Disordered TH2 immune responses may also occur in patients with atopy related gastrointestinal disorders, including IBS [12]. Studies have found that the disordered cellular immunity could involve increased intestinal mast cell infiltration in patients with IBS [13, 14]. Pearson et al. have recently reported that a patient with severe asthma and IBS treated with anti-immunoglobulin E monoclonal antibody showed improvement of both asthma and IBS symptoms [15]. Therefore, atopy may play an important role in the shared pathophysiology of asthma and IBS.

Studies have suggested that asthma and allergic disorders are associated with IBS [16–27]. However, most of these studies are based on small sample size, questionnaire, and cross-sectional or case-control studies. Bidirectional, large-scale, population-based cohort study has never been performed. The present study aimed to use Taiwan’s National Health Insurance (NHI) database to determine whether there was a bidirectional association between asthma and IBS. This dataset is a nationwide cohort dataset that has been used for various studies on asthma or IBS [28–31].

## Materials and Methods

### Data source

The Bureau of National Health Insurance (BNHI) of Taiwan has established the single-payer universal insurance system since 1995. The insurance system covers over 99.5% of the 23.74 million citizens in Taiwan (http://www.nhi.gov.tw/english/index.aspx). We used the claims data of the Longitudinal Health Insurance Database (LHID), established by the National Health Research Institutes (NHRI) of Taiwan, to conduct the present study, which included one million insured people randomly selected from all beneficiaries (n = 23.72 million) in the year 2000 registry. The LHID consisted of medical information for reimbursement from 1996 to 2011. All diseases were coded based on the International Classification of Diseases, Ninth Revision, Clinical Modification (ICD-9-CM). This study was approved by the Research Ethic Committee of China Medical University Hospital in Taiwan (CMUH-104-REC2-115). Patient records/information in the database was anonymized and de-identified prior to analysis.

### Study participants

**Fig 1:**
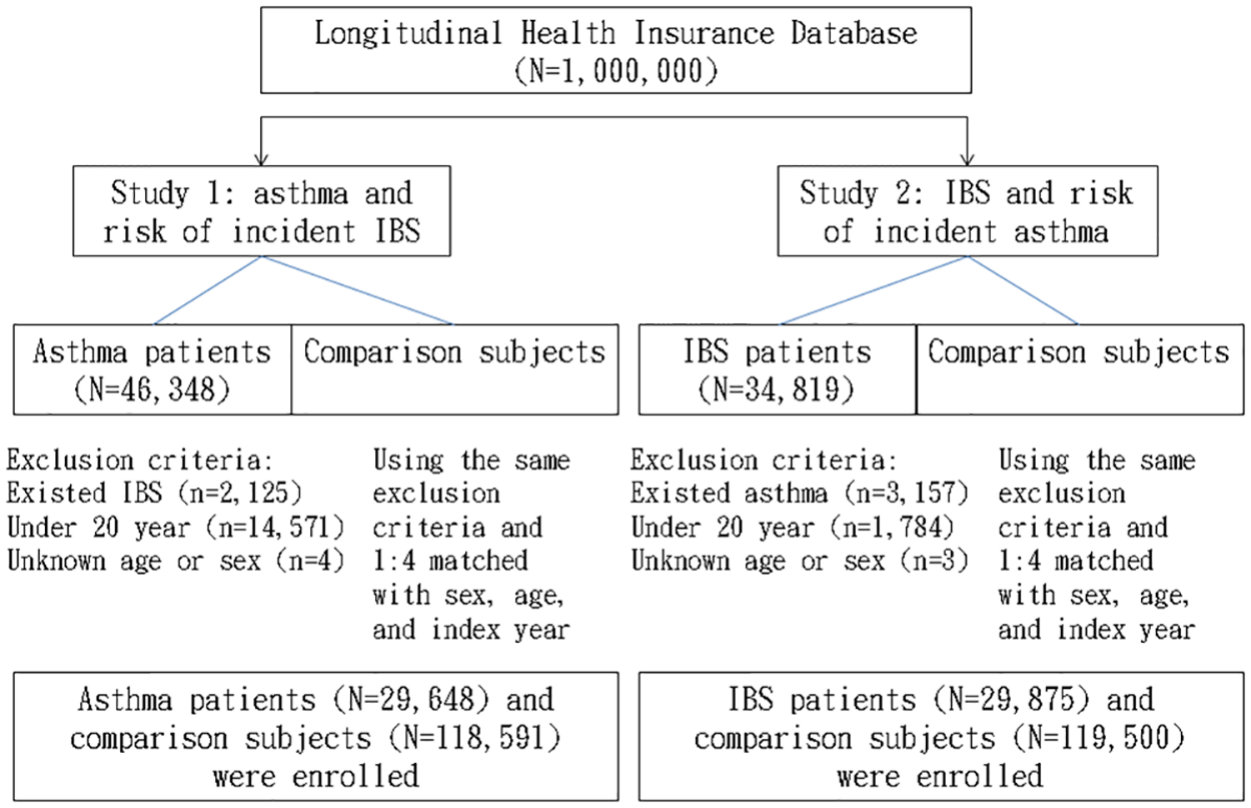
Flow chart showing selection of study subjects.

Fig 1 shows the process of identifying relevant study subjects for the two retrospective cohort studies. For Study 1, we identified patients aged ≥ 20 years with asthma diagnosis between 2000 and 2010 (ICD-9-CM code 493) for the asthma cohort. Those with asthma diagnosis before 2000 were excluded. To ensure the accuracy of asthma diagnosis, we selected only subjects who had received medications for asthma, including inhaled/systemic bronchodilator or inhaled/systemic corticosteroid into the asthma cohort. We excluded subjects with a diagnosis of IBS (ICD-9-CM code 564.1) before 2000 and those with incomplete medical information. For Study 2, patients aged ≥ 20 years with IBS diagnosis between 2000 and 2010 were identified from the same claims data. Those with IBS diagnosis before 2000 were excluded. Patients who had been diagnosed with asthma before 2000 and those with missing medical information were also excluded.

We defined the first diagnosis date as the index date for each patient. For each asthma case and each IBS case identified, four controls were selected separately as comparison cohorts for the asthma cohort and for the IBS cohort, frequency-matched by age (in 5 year spans), sex, and index year, under the same exclusion criteria.

### Outcome and relevant variables

We identified subjects with the diagnosis of IBS (for Study 1) or asthma (for Study 2) from the index date to December 31, 2011. Comorbidities included chronic obstructive pulmonary disease (COPD) (ICD-9-CM codes 496), gastro-esophageal reflux disease (GERD) (ICD-9-CM codes 530.11, and 530.81), allergic rhinitis (ICD-9-CM code 477), chronic sinusitis (ICD-9-CM code 473), atopic dermatitis (ICD-9-CM code 691), anxiety (ICD-9-CM code 300.00), depression (ICD-9-CM codes 296.2, 296.3, 300.4, 301.12, 309.0, 309.1, 311), and obesity (ICD-9-CM code 278). All comorbidities were confirmed before the index date and only patients with diagnostic codes that appeared at least twice within a year were enrolled.

### Statistical analysis

For Study 1, the distributions of categorical demographic characteristics and comorbidities were compared between the asthma cohort and the comparison cohort, and the differences were examined using the Chi-square test. The Student’s *t*-test was used to test the difference in mean ages between the two cohorts. We calculated follow-up person-years to assess the incidence density rates of IBS (per 1000 person-years) for each cohort. Univariate and multivariate Cox proportion hazard regression models were used to examine the relationship between asthma and the development of IBS. Hazard ratios (HRs) and 95% confidence intervals (CIs) were calculated. Significant variables identified in the baseline were included in the multivariate models. The proportional hazard model assumption was examined using the test of scaled Schoenfeld residuals. Results of the test revealed a significant relationship between Schoenfeld residuals for asthma and follow-up time (*p* < 0.01). In the subsequent analyses, we stratified the follow-up duration to deal with the violation of the assumption. The cumulative incidence of IBS was computed using the Kaplan–Meier method, and the differences between both cohorts were examined using the log-rank test. We used Cox proportional hazards regression analysis to measure hazard ratio of IBS by treatment [inhaled corticosteroid (ICS) vs. non-ICS]. We further used the number of emergency room (ER) visits for asthma to analyze the IBS risk associating with asthma control.

Similar data analysis procedures were performed for Study 2, and the proportional hazards model assumption was also examined. Results showed no significant relationship between Schoenfeld residuals for IBS and follow-up time (*p* = 0.96). All statistical analyses were performed using SAS 9.3 software (SAS Institute, Cary, NC, USA) for Windows. The level of significance level was set at *p* < 0.05, and the tests were 2-tailed.

## Results

### Study 1

We identified 29,648 patients in the asthma cohort and 118,591 subjects without asthma (Table 1). There were more women in both cohorts. The asthma and non-asthma cohorts were similar in age distribution; however, the asthma cohort was slightly older based on the mean age (*p* < 0.001). The patients in the asthma cohort had a higher prevalence of comorbidities than those in the non-asthma cohort (all *p* < 0.001).

**Table 1:** Comparisons in demographic characteristics and comorbidities between cohorts with and without asthma.

	Asthma				
Variables	No (N = 118591)		Yes (N = 29648)		*p*-value
	n	%	n	%	
Sex					0.99
Female	63876	53.9	15969	53.9	
Male	54715	46.1	13679	46.1	
Age, years					0.99
20–34	21560	18.2	5390	18.2	
35–49	26516	22.4	6629	22.4	
50–64	31896	26.9	7974	26.9	
≥ 65	38619	32.6	9655	32.6	
*Mean (SD)	53.7	18.1	54.2	18.1	<0.001
Comorbidity					
COPD	7728	6.52	9182	31.0	<0.001
GERD	805	0.68	479	1.62	<0.001
Allergic rhinitis	8622	7.27	8914	30.1	<0.001
Chronic sinusitis	1582	1.33	1139	3.84	<0.001
Atopic dermatitis	1528	1.29	781	2.63	<0.001
Anxiety	4916	4.15	2367	7.98	<0.001
Depression	3714	3.13	1631	5.50	<0.001
Obesity	953	0.80	519	1.75	<0.001

COPD = chronic obstructive pulmonary disease, GERD = gastroesophageal reflux disease;

Chi-square test was used to test categorical variables

*2-sample t-test was used to test mean ages.

**Fig 2:**
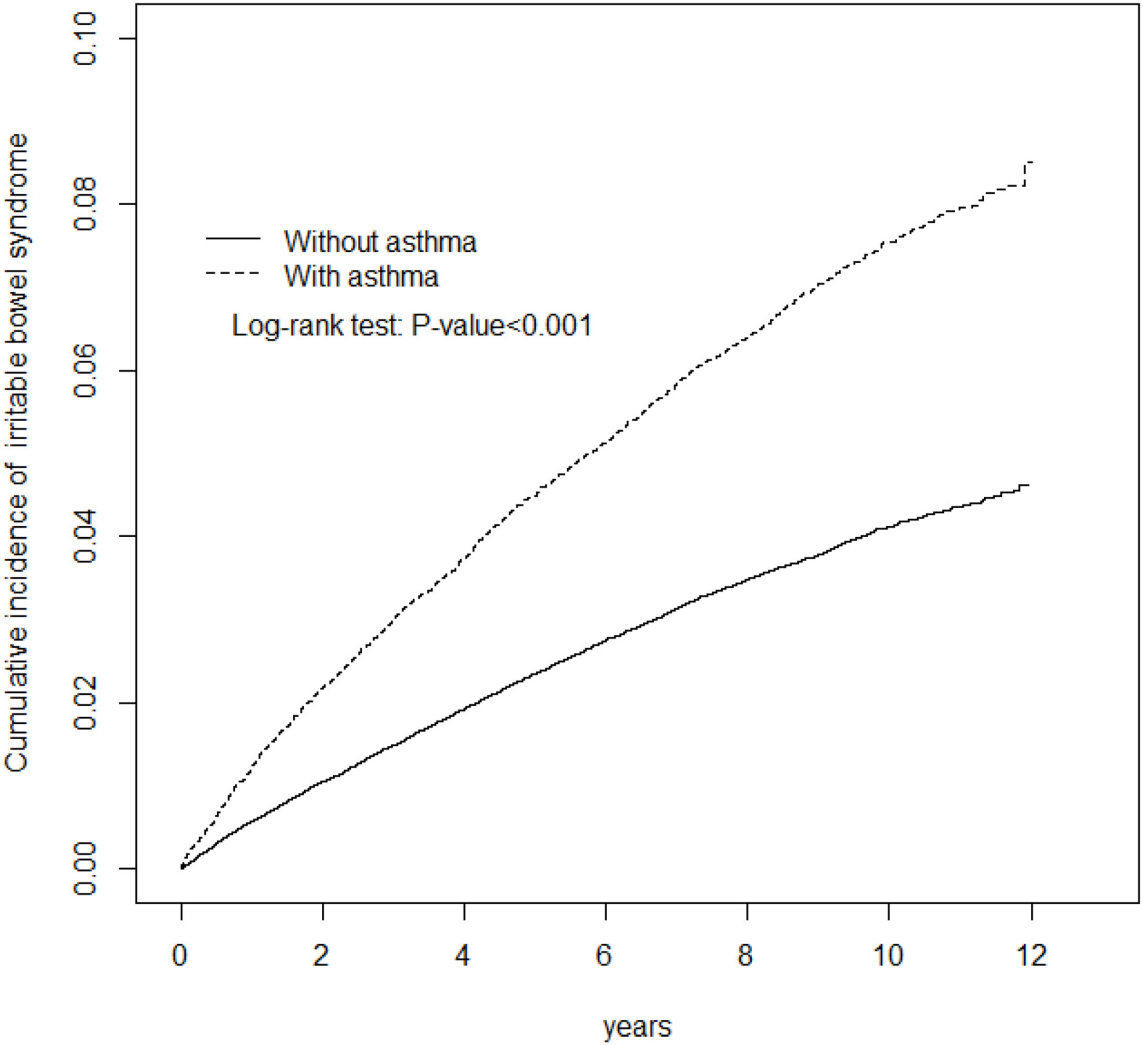
Cumulative incidence of irritable bowel syndrome for patients with (dashed line) and without (solid line) asthma.

The mean follow-up time was 6.83 (SD = 3.38) years in the asthma cohort and 6.96 (SD = 3.31) years in the non-asthma cohort (data not shown). Fig 2 shows that the cumulative incidence of IBS was 3.93% higher in the asthma cohort than in the non-asthma cohort (*p* < 0.001) by the end of follow-up.

Overall, the IBS incidence was 1.9-fold higher in the asthma cohort than in the non-asthma cohort (8.26 vs. 4.36 per 1000 person-years), with a crude HR of 1.89 (95% CI = 1.74−2.01) and an adjusted HR of 1.57 (95% CI = 1.47−1.68) (Table 2). The age-specific asthma to non-asthma adjusted hazard ratio (aHR) was the greatest for the youngest group: 2.04 (95% CI = 1.64−2.53). The aHR reduced to 1.32 (95% CI = 1.19–1.47) for the oldest group. The incidence of IBS was higher in subjects with comorbidity compared to non-comorbid subjects. The IBS incidence declined during the follow-up period in both cohorts, consistently greater in the asthma cohort than in the comparisons.

**Table 2:** Incidences and hazard ratios of irritable bowel syndrome for asthma cohort compared to non-asthma cohort by demographic characteristics, comorbidity and follow-up year.

	Asthma							
Variables	No (N = 118591)			Yes (N = 29648)			Crude HR	Adjusted HR^†^
	Event	person-years	Rate^#^	Event	person-years	Rate^#^	(95% CI)	(95% CI)
Total	3593	824817	4.36	1672	202458	8.26	1.89 (1.79–2.01)***	1.57 (1.47–1.68)***
Sex								
Female	1934	452018	4.28	900	111681	8.06	1.88 (1.74–2.04)***	1.56 (1.43–1.70)***
Male	1659	372799	4.45	772	90778	8.50	1.91 (1.75–2.08)***	1.59 (1.45–1.75)***
Age, years								
20–34	287	156840	1.83	171	39844	4.29	2.35 (1.95–2.84)***	2.04 (1.64–2.53)***
35–49	638	198936	3.21	376	49179	7.65	2.38 (2.10–2.71)***	1.84 (1.59–2.13)***
50–64	1150	231353	4.97	540	56687	9.53	1.92 (1.73–2.12)***	1.59 (1.42–1.79)***
≥ 65	1518	237687	6.39	585	56748	10.3	1.61 (1.46–1.77)***	1.32 (1.19–1.47)***
Comorbidity^‡^								
No	2547	689233	3.70	606	91041	6.66	1.81 (1.66–1.98)***	1.85 (1.69–2.02)***
Yes	1046	135583	7.71	1066	111417	9.57	1.27 (1.16–1.38)***	1.33 (1.22–1.45)***
Follow-up year								
<2	671	116860	5.74	354	29124	12.2	2.12 (1.86–2.41)***	1.60 (1.39–1.85)***
2−3	994	221962	4.48	487	55251	8.81	1.97 (1.77–2.19)***	1.69 (1.49–1.90)***
4−5	798	219986	3.63	346	55015	6.29	1.73 (1.53–1.97)***	1.45 (1.26–1.67)***
≥ 5	1130	311800	3.62	485	75862	6.39	1.77 (1.59–1.97)***	1.54 (1.37–1.74)***

Crude HR = relative hazard ratio, CI = confidence interval;

Rate^#^, incidence rate per 1000 person-years;

^†^ Model was adjusted for age, sex, and comorbidities of COPD, GERD, allergic rhinitis, chronic sinusitis, atopic dermatitis, anxiety, depression, and obesity;

^‡^ Patients with any comorbidity of COPD, GERD, allergic rhinitis, chronic sinusitis, atopic dermatitis, anxiety, depression, and obesity were defined as the comorbidity group;

*** *p* < 0.001.

Table 3 shows the effectiveness of treating. The IBS incidence was lower in patients with ICS treatment than those without the treatment, but not significant (aHR: 0.93, 95% CI = 0.84–1.03). Table 4 shows that the hazard of IBS increased with the frequency of ER visit, to an aHR of 20.7 (95% CI = 15.6–27.4) for those with more than twice a year of ER visits (*p* for trend < 0.0001), compared with the comparison cohort.

**Table 3:** Cox proportional hazards regression analysis measured hazard ratio of irritable bowel syndrome for asthma patients by treatment.

					Crude HR	Adjusted HR^†^
Variables	N	Events	PY	Rate^#^	(95% CI)	(95% CI)
Treatments of asthma						
Non-ICS user	17039	965	112256	8.60	1 (Reference)	1 (Reference)
ICS user	12609	707	90202	7.84	0.92 (0.84–1.02)	0.93 (0.84–1.03)

Crude HR = relative hazard ratio, CI = confidence interval, ICS = inhaled corticosteroids;

Rate^#^, incidence rate per 1000 person-years;

^†^ Model was adjusted for age, sex, and comorbidities of COPD, GERD, allergic rhinitis, chronic sinusitis, atopic dermatitis, anxiety, depression, and obesity.

**Table 4:** Hazard ratios of irritable bowel syndrome associated with mean number of annual emergency room visits for asthma.

	Events	Crude HR (95% CI)	Adjusted HR^†^ (95% CI)
Non-asthma	3593	1 (Reference)	1 (Reference)
Times of emergency room visit			
<1	1591	1.81 (1.71–1.92)***	1.51 (1.42–1.62)***
1−2	30	7.97 (5.56–11.4)***	6.23 (4.34–8.95)***
>2	51	30.3 (22.9–39.9)***	20.7 (15.6–27.4)***
*p* for trend		<0.001	<0.001

HR = hazard ratio, CI = confidence interval;

^†^ Model was adjusted for age, sex, and comorbidities of COPD, GERD, allergic rhinitis, chronic sinusitis, atopic dermatitis, anxiety, depression, and obesity;

*** *p* < 0.001.

### Study 2

Table 5 shows that both the IBS and non-IBS cohorts were dominated by women (52.8%), and 31% of the subjects were aged 35–49 years old. The mean age of the IBS cohort was slightly higher than that of the non-IBS, but significant. Comorbidities were also more prevalent in the IBS cohort (all *p* < 0.001).

**Table 5:** Comparisons in demographic characteristics and comorbidities between cohort with and without irritable bowel syndrome.

	Irritable bowel syndrome				
Variables	No (N = 119500)		Yes (N = 29875)		*p*-value
	n	%	n	%	
Sex					0.99
Female	63144	52.8	15786	52.8	
Male	56356	47.2	14089	47.2	
Age, years					0.99
20–34	23944	20.0	5986	20.0	
35–49	37064	31.0	9266	31.0	
50–64	31828	26.6	7957	26.6	
≥ 65	26664	22.3	6666	22.3	
*Mean (SD)	49.9	(16.9)	50.4	(16.7)	<0.001
Comorbidity					
COPD	7591	6.35	3437	11.5	<0.001
GERD	848	0.71	1318	4.41	<0.001
Allergic rhinitis	9966	8.34	5107	17.1	<0.001
Chronic sinusiti	1734	1.45	947	3.17	<0.001
Atopic dermatitis	1608	1.35	640	2.14	<0.001
Anxiety	4601	3.85	4210	14.1	<0.001
Depression	3318	2.78	2593	8.68	<0.001
Obesity	1055	0.88	386	1.29	<0.001

COPD = chronic obstructive pulmonary disease, GERD = gastroesophageal reflux disease;

Chi-square test was used to test categorical variables

*2-sample t-test was used to test mean ages.

**Fig 3:**
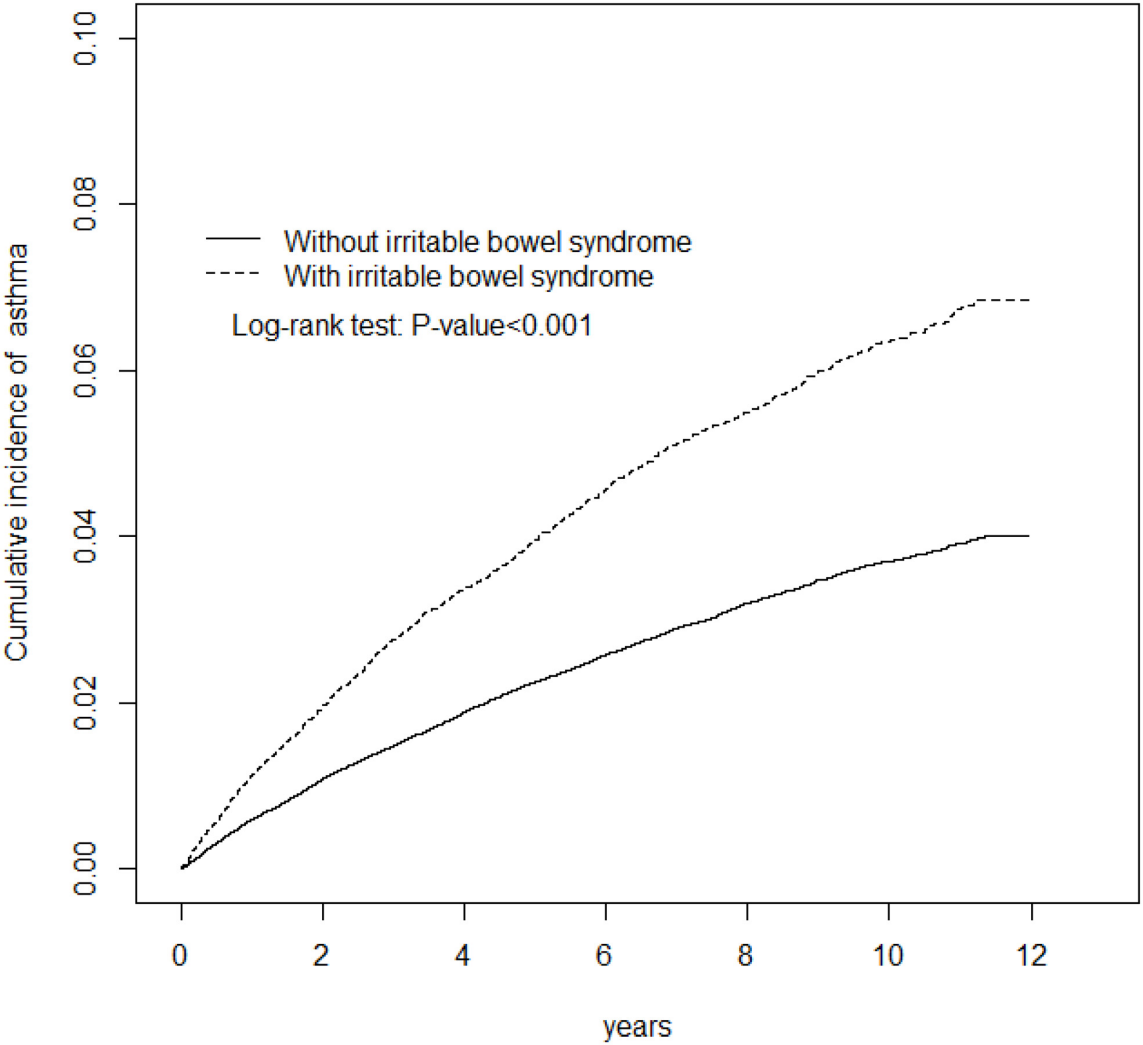
Cumulative incidence of asthma for patients with (dashed line) and without (solid line) irritable bowel syndrome.

After 12 years of follow-up, the cumulative incidence of asthma was 2.83% higher in the IBS cohort than in the non-IBS cohort (*p* < 0.001, Fig 3). The overall incidence of asthma was 1.8-fold higher in the IBS cohort than in the non-IBS cohort (7.09 vs. 4.03 per 1000 person-years), with an aHR of 1.54 (95% CI = 1.44−1.64) (Table 6). The sex-specific and age-specific IBS to non-IBS aHRs were all significant for women and men and for all age groups. Comorbidities increased the incidence of asthma in both cohorts, with the aHR (IBS cohort to the non-IBS cohort) stronger for those without comorbidity. The asthma incidence declined over time in both cohorts, but the trend of changes in the aHR (IBS cohort to the non-IBS cohort) was limited.

**Table 6:** Incidences and hazard ratios of asthma for irritable bowel syndrome cohort compared to non-irritable bowel syndrome cohort by demographic characteristics, comorbidity, and follow-up year.

	Irritable bowel syndrome							
Variables	No (N = 119500)			Yes (N = 29875)			Crude HR	Adjusted HR^†^
	Event	person-years	Rate^#^	Event	person-years	Rate^#^	(95% CI)	(95% CI)
Total	3260	809513	4.03	1444	203689	7.09	1.76 (1.66−1.88)***	1.54 (1.44−1.64)***
Sex								
Female	1828	436904	4.18	812	109511	7.41	1.77 (1.63−1.93)***	1.58 (1.45−1.72)***
Male	1432	372609	3.84	632	94178	6.71	1.75 (1.59−1.92)***	1.49 (1.35−1.64)***
Age, years								
20–34	281	170951	1.64	139	43682	3.18	1.94 (1.59−2.38)***	1.65 (1.33−2.04)***
35–49	575	267825	2.15	341	67064	5.08	2.37 (2.07−2.71)***	2.00 (1.74−2.31)***
50–64	1035	214844	4.82	456	53055	8.59	1.78 (1.60−1.99)***	1.54 (1.38−1.73)***
≥ 65	1369	155893	8.78	508	39889	12.7	1.46 (1.32−1.62)***	1.31 (1.18−1.45)***
Comorbidity^‡^								
No	2065	676473	3.05	659	128560	5.13	1.69 (1.55−1.85)***	1.72 (1.57−1.87)***
Yes	1195	133039	8.98	785	75130	10.5	1.19 (1.09−1.30)***	1.25 (1.14−1.37)***
Follow-up year								
<2	701	118008	5.94	332	29519	11.3	1.89 (1.66−2.16)***	1.64 (1.44−1.88)***
2−3	967	221650	4.36	452	55419	8.16	1.87 (1.67−2.09)***	1.62 (1.44−1.82)***
4−5	699	220559	3.17	276	55226	5.00	1.58 (1.37−1.81)***	1.43 (1.23−1.65)***
≥ 5	893	297853	3.00	384	75441	5.09	1.70 (1.51−1.92)***	1.51 (1.33−1.71)***

Crude HR = relative hazard ratio, CI = confidence interval;

Rate^#^, incidence rate per 1000 person-years;

^†^ Model was adjusted for age, sex, and comorbidities of COPD, GERD, allergic rhinitis, chronic sinusitis, atopic dermatitis, anxiety, depression, and obesity;

^‡^ Patients with any comorbidity of COPD, GERD, allergic rhinitis, chronic sinusitis, atopic dermatitis, anxiety, depression, and obesity were defined as the comorbidity group;

*** *p* < 0.001.

## Discussion

This population-based cohort study demonstrated a bidirectional association between asthma and IBS. We found that there are a significantly higher risk of IBS in patients with asthma than in the general population, and a significantly increased risk of asthma in patients with IBS than in the general population.

In recent decades, several studies have investigated the relationship between asthma and IBS. Kennedy et al. reported earlier an independent association between IBS and bronchial hyper-responsiveness [16]. Subsequently, several small-scale case-control studies reported that the prevalence rates of IBS were higher in patients with asthma (27.5–41.3%) than in non-asthma subjects (7.93–20.8%) [17–21]. Huerta et al. reported a slightly increased risk of IBS in asthma patients compared to the general population (2.5 vs. 2.0 per 1000 person-years, RR: 1.3, 95% CI = 1.1–1.5) in a large UK-based population cohort [22]. They also found the use of oral steroids in asthma patients could reduce the risk of IBS. Another large-scale study in the US by Cole et al. reported a 20% increase in the incidence of IBS among asthma patients, but they failed to find the effect of oral steroids among these patients [23]. Our study also failed to show a significant effectiveness of ICS treatment in reducing the IBS risk for asthma patients. The inconsistent findings in the ICS medications propose the need for additional investigations.

On the other hand, Yazar et al. found in a case-control study that the prevalence of asthma was much greater in IBS cases than in healthy controls (15.8 vs. 1.45%) based on medical history, clinical features, and the results of pulmonary function test [24]. In another case-control analysis using medical records of 30,000 patients in primary care settings, Jones et al. found patients with IBS were more prevalent with asthma history than non-IBS subjects (15.0 vs. 11.0%) [6]. In a large community survey, Amra et al. also found a near 3-fold higher prevalence of asthma in IBS patients than in non-IBS subjects (9.5% vs. 3.3%) [25]. These findings are consistent with our cohort study finding: IBS patients are at an elevated risk of developing asthma. The mechanisms behind the bidirectional association between asthma and IBS, or concomitant factors existing in these two diseases are largely unknown. Atopy may play an important role in the association. A questionnaire study has found that patients with atopic manifestations, such as allergic rhinitis, allergic eczema, and asthma, are near 3-time more likely to have IBS [7]. Individuals with hypersensitivity to food and pollen may associate with the manifestation of IBS [32, 33]. The underlying causes of inflammatory conditions can also produce respiratory and gastrointestinal symptoms, as well as smooth muscle hyperactivity [7, 23]. Other shared risks and comorbid conditions, such as smoking, GERD, mood disorders and obesity may also play a role. In addition, socioeconomic level, education, occupation, residence area and nutrition status may potentially confound both diseases, which cannot be totally corrected in this study.

It is important to note that the IBS diagnosis is criteria based, most using the Rome III criteria, which can be challenging due to overlap with other organic conditions [34–36]. The potential conditions include celiac disease, chronic small intestinal bacterial overgrowth, bile acid diarrhea, malabsorption because of exocrine pancreatic insufficiency, and inflammatory bowel disease etc. There is considerable heterogeneity in both sensitivity and specificity among studies. The sensitivity and specificity of the IBS diagnosis can be improved by verification with the data of laboratory tests, especially results of screening tests for inflammation and blood in stools [36]. However, the information of laboratory tests was not available, we could not perform the validation in the present study. Among these conditions, we found that celiac disease and inflammatory bowel disease were associated with asthma [37–39]. Therefore, any misclassifications may influence our results as well.

In Study 1, our findings are compatible with the well-known concept that the prevalence of comorbidities such as COPD, GERD, allergic rhinitis, chronic sinusitis, atopic dermatitis, anxiety, depression, and obesity are significantly higher in patients with asthma than in controls. Asthma patients with comorbidities had a higher incidence of IBS than those without comorbidities and non-asthma subjects with comorbidities. This may be partly explained by the fact that patients with asthma and comorbid conditions may require multiple medical visits and are at a greater risk of receiving an additional diagnosis. In addition, our study revealed that the IBS risk increased proportionately with the number of annual ER visits for asthma. Therefore, a higher incident IBS rate may be partly associated with Berkson’s bias [40, 41]. Similarly, in Study 2, the prevalence rates of comorbidities, including COPD, GERD, allergic rhinitis, chronic sinusitis, atopic dermatitis, anxiety, depression, and obesity, were also significantly higher in patients with IBS than in the controls. IBS patients with any of these comorbidities had a higher incidence of asthma than those without comorbidities and non-IBS subjects with comorbidities. Thus, a higher incident asthma rate may also be partly associated with Berkson’s bias.

The strength of this study is the use of a longitudinal population-based evaluation for the bidirectional relationship between asthma and IBS. It is generally costly to conduct a population-based prospective cohort study, in which loss to follow-up is problematic after years of follow-up. Therefore, using insurance claims data to conduct a retrospective cohort study is a timely economical alternative. However, there are several limitations to be considered about interpreting the study results. First, this study used the ICD-9-CM algorithm to define diseases based the clinical performance of physicians. However, the insurance authority has established an ad hoc committee to monitor the accuracy of claims data to prevent violation. In addition, we selected only subjects with repeated coding to increase the validity and accuracy of the diagnoses. Second, NHIRD does not provide detailed information on occupation, smoking habits, body mass index, diet preference, environmental exposure, or family history, although these are potential confounding factors. Our data analysis used the comorbidity variables of COPD and obesity as part of the controlling variables to substitute smoking and sociodemographic status. In addition, relevant clinical variables, such as pulmonary function tests, serum laboratory data, or imaging results, were unavailable for diagnosis validation. However, the significant bidirectional relationship between asthma and IBS has been approved in our data. The dose response association further show that the relationship is likely real.

## Conclusion

This study suggests a bidirectional association between asthma and IBS. The risk of incident IBS for asthma patients is slightly greater than the risk of incident asthma for IBS patients. The association could be clinical and pathophysiological importance. Both asthma and IBS may share a similar pathophysiology underlying this association instead of a causal relationship between the two disorders. Our data suggest that there is a need to monitor asthma patients for the potential of developing IBS, and vice versa.
